# BRED: A Simple and Powerful Tool for Constructing Mutant and Recombinant Bacteriophage Genomes

**DOI:** 10.1371/journal.pone.0003957

**Published:** 2008-12-17

**Authors:** Laura J. Marinelli, Mariana Piuri, Zuzana Swigoňová, Amrita Balachandran, Lauren M. Oldfield, Julia C. van Kessel, Graham F. Hatfull

**Affiliations:** Pittsburgh Bacteriophage Institute and Department of Biological Sciences, University of Pittsburgh, Pittsburgh, Pennsylvania, United States of America; Pacific Northwest National Laboratory, United States of America

## Abstract

Advances in DNA sequencing technology have facilitated the determination of hundreds of complete genome sequences both for bacteria and their bacteriophages. Some of these bacteria have well-developed and facile genetic systems for constructing mutants to determine gene function, and recombineering is a particularly effective tool. However, generally applicable methods for constructing defined mutants of bacteriophages are poorly developed, in part because of the inability to use selectable markers such as drug resistance genes during viral lytic growth. Here we describe a method for simple and effective directed mutagenesis of bacteriophage genomes using Bacteriophage Recombineering of Electroporated DNA (BRED), in which a highly efficient recombineering system is utilized directly on electroporated phage DNA; no selection is required and mutants can be readily detected by PCR. We describe the use of BRED to construct unmarked gene deletions, in-frame internal deletions, base substitutions, precise gene replacements, and the addition of gene tags.

## Introduction

Bacteriophage genetics have played central roles in the development of bacterial genetics, the elucidation of the genetic code, and the birth of biotechnology[1]. Phages continue to be of interest for three main reasons. First, they are rich and powerful toolboxes for the development of genetic systems in genetically naive bacterial species[2]. Second, they play key roles in food commerce, such as in the dairy industry[3] and in the control of *Listeria* contamination[4]. Finally, their high genetic diversity, enormous abundance, and richness in genetic novelty[5] suggest that phages represent the largest reservoir of unexplored genetic information in the biosphere[6], [7].

Sophisticated methods for mutant isolation and mutational mapping by recombination have been described for a few prototype phages such as λ, T4, and T7, and although in principle these could be applied to other phages, there are few examples of this. Furthermore, broadly applicable methods for efficient construction of defined mutations in phage genomes are lacking, in sharp contrast to the range of approaches that have been described for targeted mutagenesis of bacterial chromosomes[8], [9]. One example is a technique known as recombineering or genetic engineering mediated by recombination proteins[8], [10]. This was developed in *Escherichia coli* and other Gram-negative organisms using the bacteriophage λ Red recombination proteins, Exo and Beta, which efficiently promote homologous recombination between linear DNA substrates and homologous targets in the bacterial chromosome[8], [11]–[14]. The Rac prophage RecE and RecT proteins function similarly and have also been exploited for mutant construction[15], [16]. These systems allow the mutagenesis of lysogenic phages through prophage recombineering[17] as well as mutagenesis of lytically replicating phages[18]. However, the efficiency of recombineering in lytic growth is low, and antibiotic resistance cannot typically be used for mutant selection.

Bacteriophages have played important roles in the development of genetic systems for *Mycobacterium tuberculosis*, a slow-growing bacterium that causes human tuberculosis[19], [20]. More than 30 mycobacteriophage genomes have been sequenced, revealing them to be genetically diverse, replete with novel sequences, and having mosaic genomic architectures[21], [22]. Recombinant mycobacteriophages have been constructed using shuttle phasmids – chimeras that replicate as large plasmids in *E. coli* and as viruses in mycobacteria[23] – and by recombination with plasmids[24]. Shuttle phasmids are amenable to mutagenesis by recombineering in *E. coli* [25], but the relatively large size of mycobacteriophage genomes restricts the number of phages for which this is applicable[22], and recombination from plasmids is tedious and inefficient[24].

Here we describe a novel approach – Bacteriophage Recombineering of Electroporated DNA (BRED) – for simple and efficient construction of targeted bacteriophage mutants. We demonstrate that BRED can be used for the construction of unmarked deletions of both essential and non-essential genes, in-frame internal deletions, point mutations and nonsense mutations, the addition of gene tags, and the precise insertion of foreign genes. This technique works in all mycobacteriophages that we have tested and should be generally applicable to phages of other hosts in which recombineering systems are available.

## Results

### The BRED Strategy

**Figure 1:**
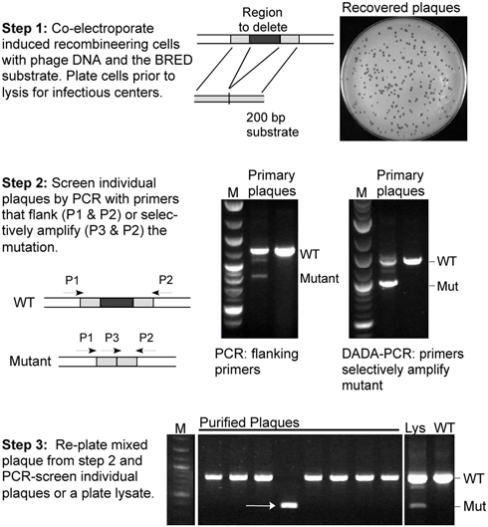
A simple three-step method for constructing bacteriophage mutants using BRED. In the first step, induced electrocompetent *M. smegmatis* mc^2^155 cells containing the recombineering plasmid pJV53 are co-transformed with phage DNA (50–100 ng) and the recombineering substrate (50–500 ng); a 200 bp PCR-generated dsDNA substrate containing a centrally located mutation is typically used. Cells are recovered for ∼2 hours and plated as top agar lawns with *M. smegmatis* plating cells. The second BRED step involves screening individual plaques by PCR with primers that either flank the mutation and/or with primers that selectively amplify the mutation and can detect fewer molecules. In the case of deletions, insertions or most gene replacements, mutant bands are differentially sized and are distinguishable from wild-type. In the final step a mixed plaque detected in step 2 is diluted and re-plated for isolated plaques that are then screened again by PCR. Alternatively, a lysate (LYS) generated by pooling many plaques (∼1000–5000) can be analyzed by PCR. If the mutant is viable then the mutation is present in the lysate, whereas if the gene is essential, the mutation is no longer present.

BRED takes advantage of the previously described mycobacterial recombineering system, in which expression of the RecE/RecT-like proteins gp60 and gp61 of mycobacteriophage Che9c confers high levels of homologous recombination[26]. Chromosomal gene replacements can be constructed using double-stranded DNA (dsDNA) substrates with >500 bp of homology[27], and point mutations are made using single-stranded DNA (ssDNA) substrates with >45 bp homology[28]. The targeting substrates are introduced by electroporation, and while this is sufficiently efficient for mutant recovery, the proportion of total cells that take up DNA is small (∼0.1%). In BRED, phage DNA template and a targeting substrate are co-electroporated into *Mycobacterium smegmatis* cells that have been induced for recombineering functions (Fig. 1). These are plated in an infectious center assay, such that plaques are derived from individual cells that have taken up phage DNA and converted it into infectious particles. Because a high proportion of cells that take up phage DNA also take up substrate DNA, and because of the elevated recombination levels, a substantial proportion of the recovered plaques contain the desired mutant genome in addition to wild-type phage DNA. While the proportion of mutant genomes varies considerably among individual plaques, mutants can be readily recovered and are identified by PCR analysis of individual plaques that arise from re-plating a mixed population (Fig. 1). If the mutant phage is non-viable, mutants can nevertheless be recovered by complementation or suppression, as described below.

### Use of BRED to construct an internal deletion of the Giles tape measure gene

**Figure 2:**
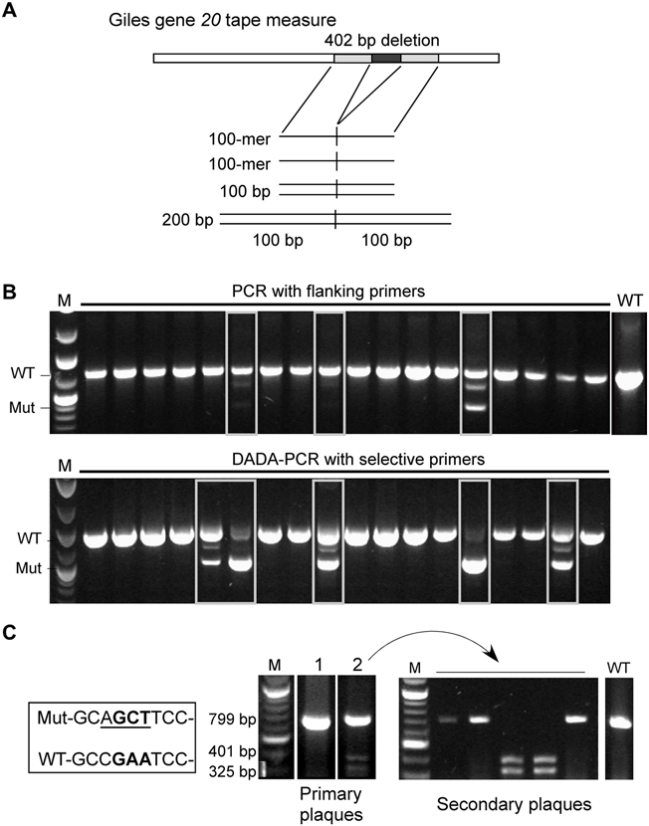
Use of BRED to construct internal deletions and point mutations in the tape measure gene of mycobacteriophage Giles. a. Schematic illustration of substrates used for recombineering. b. *M. smegmatis* mc^2^155∶pJV53 cells were co-transformed with 50 ng Giles DNA and a 200 bp dsDNA deletion substrate (300 ng), plaques were recovered and PCR-screened with flanking primers (top gel) or selective primers (lower gel) as indicated. Samples containing mutant genomes in addition to wild-type DNA (boxed lanes) are detected by both methods, but are detected more frequently by selective DADA-PCR. Pools containing the deletion were re-plated, and plaques were screened for pure populations of mutant phage by flanking PCR (not shown). c. Base substition mutations, which change a glutamate (GAA) codon to an alanine (GCT) and incorporate an AluI restriction site for screening (boxed sequence), were engineered into Giles gene *20* using a 70 bp dsDNA substate (200 ng) co-transformed into mc^2^155∶pJV53 cells with 50 ng Giles DNA. Two primary plaques from the initial screening are shown (#1, #2) one of which (#2) has products of Alu I cleavage. Secondary plaques recovered from the mixed plaque were screened, and the wild-type and two mutant plaques are shown.

To evaluate the BRED strategy, we attempted to construct a deletion derivative of mycobacteriophage Giles[29] in which a central 402 bp portion of the tape measure gene is removed (Fig. 2A). Initially, we used a 200 bp dsDNA substrate that has 100 bp of homology to the Giles genome on each side of the deleted region. Co-electroporation of 50 ng of Giles DNA and 200 ng of substrate yielded ∼100 plaques, and of the 29 that were tested by PCR with primers flanking the deletion, all were found to contain wild-type DNA; however, three also had detectable levels of the mutant allele (Table 1). A 100 bp dsDNA substrate and 100-nucleotide ssDNA oligonucleotides were also tested, although none gave higher proportions of mixed plaques than the 200 bp dsDNA substrate (Table 1). Similar proportions of mutant-containing plaques were observed using substrates that generate a 717 bp in-frame deletion in Giles gene *20*, and increasing the amount of 200 bp dsDNA substrate did not substantially alter the proportion of mixed plaques detected by flanking PCR (Table 1). Plaques containing the deletion were not recovered from control cells lacking pJV53 (data not shown), indicating that mutagenesis is dependent on the Che9c recombineering functions. These data show that mutant-containing plaques can be recovered at a remarkably high efficiency (10–15%) and can be readily identified in the absence of any selection (Table 1).

**Table 1:** BRED frequencies for constructing deletions in the tape measure gene of mycobacteriophage Giles.

Deletion (bp)^1^	Substrate^2^	Amount	# Mixed plaques/Total analyzed (%)^3^	
			Flanking primer PCR	DADA-PCR
402 bp	LJM119-100 nt	200 ng	1/29 (3.4%)	NT
402 bp	LJM120-100 nt	200 ng	0/29	NT
402 bp	dsDNA-100 bp	200 ng	1/19 (5.3%)	NT
402 bp	dsDNA-200 bp	200 ng	3/29 (10.3%)	NT
717 bp	LJM123-100 nt	150 ng	0/18	NT
717 bp	LJM124-100 nt	150 ng	0/18	NT
717 bp	dsDNA-100 bp	150 ng	1/18 (5.6%)	NT
717 bp	dsDNA-200 bp	150 ng	4/18 (22.2)	NT
402 bp^4^	dsDNA-200 bp	100 ng	6/36 (16.7%)	12/36 (33.3%)
402 bp^4^	dsDNA-200 bp	200 ng	6/36 (16.7%)	12/36 (33.3%)
402 bp^4^	dsDNA-200 bp	300 ng	6/36 (16.7%)	14/36 (38.9%)
402 bp^4^	dsDNA-200 bp	400 ng	4/36 (11.1%)	15/36 (41.7%)

1 Mutants generated have in-frame deletions in Giles gene *20*; deletion sizes in base pairs (bp).

2 Substrates were either oligonucleotides (name-length) or dsDNA as indicated.

3 Plaques recovered from electroporation analyzed by PCR as indicated; NT, not tested.

4 Substrate also incorporates point mutations; frequencies reflect deletion formation only.

Since all of the recovered plaques contain wild-type phage DNA, recombination presumably occurs only after DNA replication has begun. Thus, the ratio of mutant to wild-type genomes in the recovered plaques is expected to vary greatly and to fluctuate depending on when recombination occurs. Detection of plaques containing mutant alleles may therefore also vary depending on the sensitivity of the PCR method employed. To test this, we re-analyzed 144 primary plaques recovered from a BRED experiment (36 each from four experiments with different amounts of substrate) and compared the number of mixed plaques detected by flanking primer PCR with those detected by a Deletion Amplification Detection Assay (DADA)-PCR. This uses a primer with a 3′ end annealing across the junction created by the deletion and preferentially amplifies the mutant template, similar to the previously described Mismatch Amplification Mutation Assay (MAMA)-PCR assay[30]. Approximately twice as many mixed plaques were identified by DADA-PCR as by flanking primer PCR (Fig. 2B, Table 1). The overall efficiency of BRED mutagenesis is therefore reflected in two values; the number of plaques containing detectable levels of mutant DNA, and the proportion of mutant genomes within those plaques.

Identification of homogenously mutant phage derivatives was accomplished by re-plating serial dilutions of mixed plaques and screening individual plaques by PCR. The proportion of mutant plaques was expected to be directly related to the ratio of mutant to wild-type genomes in the primary mixed plaque. To recover the 402 bp gene *20* deletion mutant, ten mixed plaques were picked and re-plated and single plaques tested by PCR; at least one pure mutant was identified in seven of these, although at greatly varying frequencies (1/15, 2/15, 3/16, 10/15, 2/17, 2/24, 2/15; 19.8% average). The three mixed plaques from which we could not isolate the mutant (after testing 25–27 individual plaques) had only barely detectable levels of the mutant in the primary plaque when examined using flanking primer PCR. The high sensitivity of DADA-PCR is thus a double-edged sword; it enables identification of mixed plaques containing lower proportions of the mutant, but recovering the mutant from secondary plating may require extensive screening. In contrast, a mixed plaque that is readily identified by flanking primer PCR is likely to require the screening of far fewer secondary plaques.

We also tested whether we could introduce base changes in Giles gene *20* that confer an amino acid substitution. A 70 bp dsDNA substrate was used that alters two adjacent bases and introduces an Alu I restriction site (Fig. 2C); 18 primary plaques were screened by PCR and Alu I digestion, one of which was clearly a mixed plaque (Fig. 2C). Two pure mutant samples were identified by screening ten plaques from secondary plating, indicating that point mutations as well as deletions can be readily introduced using BRED. We further examined the linkage of multiple mutations within a single substrate by using a 200 bp dsDNA substrate, similar to that used for the Giles gp20 deletion, but which contains the same base substitutions described above, 57 bp to the right of the deletion endpoint. Seven primary plaques containing the deletion were identified by flanking PCR and re-plated. Individual secondary plaques were then screened for both the deletion and the Alu I site. In four of the seven mixed plaques tested, all of the deletion mutants identified contained the Alu I site, whereas for the other three, none of the deletion mutants contained the site. The mechanism by which the mutations become unlinked is unclear, especially since mycobacteria lack a mismatch repair system and are reported to be functionally mismatch repair defective[31].

### Construction of phage mutants defective in essential genes

**Figure 3:**
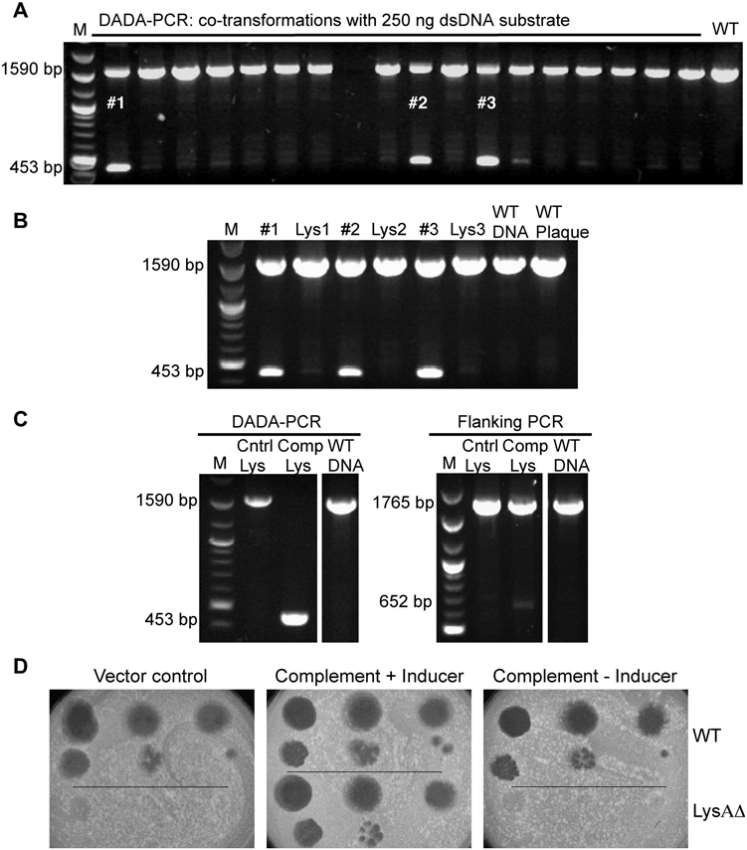
Use of BRED to construct a deletion mutant of the essential Giles *lysA* gene. a. A 200 bp dsDNA substrate designed to introduce a 1173 bp deletion in Giles *lysA* was co-electroporated with Giles DNA into recombineering cells, and individual plaques were tested using DADA-PCR. Three mixed plaques are indicated (#1, #2, #3). b. The three mixed-plaques marked in part a were re-plated, and lysates were generated from plates containing 1000–5000 plaques. Each lysate (Lys) and the original mixed plaque were analyzed by DADA-PCR with loss of the mutant in the lysate suggesting that *lysA* is an essential gene. c. Mixed plaque #3 was re-plated on either *M. smegmatis* (Cntrl) or a recombinant strain expressing the Corndog *lysA* gene (Comp), and lysates were harvested from plates containing ∼2000 plaques. Screening by DADA-PCR or flanking PCR shows that the mutant is propagated in the complementing but not in the wild-type strain. d. The LysAΔ mutant was purified from the complementation strain and confirmed by PCR and DNA sequencing. Serial dilutions of mutant (lower parts) and wild-type phage (upper parts) were spotted onto lawns seeded with either the vector control cells or the complementation strain in the presence or absence of acetamide, as indicated.

Although the lysis system of mycobacteriophages is not well understood, we reasoned that genes involved in lysis are likely to be required for plaque formation. We therefore tested whether we could construct a mutant in which the lysin A (*lysA*) gene is deleted and recover it by complementation. A 200 bp dsDNA substrate designed to introduce a 1,173 bp deletion into the Giles *lysA* gene (*31*) was co-electroporated with Giles phage DNA. Plaques were recovered and examined by flanking and DADA-PCR (Fig. 3A) revealing mixed plaques at frequencies of 5–12% and 20–36%, respectively (Table 2). Although we predicted *lysA* to be essential, the mutant presumably grows in the mixed plaque through assistance of wild-type helper phage. To demonstrate Giles *lysA* essentiality, three separate mixed plaques were re-plated, and 1000–5000 individual plaques from each (all derived from a single particle) were harvested to generate secondary lysates. For all three mixed plaques, the mutant could not be identified by DADA-PCR in the lysate (Fig. 3B), suggesting strongly that *lysA* is indeed essential for Giles propagation.

**Table 2:** BRED frequencies for the construction of a Giles *lysA*Δ mutant.

Deletion (bp)	Substrate	Amount	# Mixed plaques/Total analyzed (%)^1^	
			Flanking primer PCR	DADA-PCR
1173 bp	dsDNA-200 bp	50 ng	2/35 (5.7%)	7/34 (20.6%)
1173 bp	dsDNA-200 bp	250 ng	3/35 (8.6%)	10/35 (28.6%
1173 bp	dsDNA-200 bp	500 ng	4/33 (12.1%)	13/36 (36.1%)

1 Plaques recovered from electroporation analyzed by PCR as indicated.

Therefore, to recover and propagate the *lysA* mutant, a complementing strain was constructed in which the *lysA* gene of phage Corndog is under control of the inducible acetamidase promoter[32]. Preliminary experiments showed that Corndog *lysA* can be expressed from an induced acetamidase promoter without cell death, unlike other mycobacteriophage *lysA* genes we have tested. A mixed plaque was then plated onto complementing and control strains, and secondary lysates prepared from the harvesting of about 2000 plaques from each strain were tested both by flanking primer and DADA-PCR. Mutant DNA could readily be identified in the lysate from the complementing strain but not from the control (Fig. 3C), indicating that Corndog *lysA* can complement the Giles mutant. Individual plaques recovered on the complementing strain were tested for dependence on complementation, and of 100 plaques examined, one was identified that does not grow on the control strain. The presence of the deletion was confirmed by PCR, and the purified *lysA* mutant was further shown to form plaques only on the complementing strain, and only in the presence of inducer (Fig. 3D). As expected, revertants of the *lysA* deletion mutant were not detected after plating up to 10^8^ particles on a control strain.

In a separate experiment, we also tested whether a nonsense mutation could be introduced into the *lysA* gene of TM4. TM4 DNA was co-electroporated with a 100 bp dsDNA substrate, and plaques recovered on *M. smegmatis* plating cells expressing a nonsense suppressor[33]. Mixed plaques containing the mutation were identified by MAMA-PCR, and from one of these, individual plaques containing the mutation were isolated on the suppressor strain (see Table 3).

**Table 3:** Summary of mycobacteriophage mutants generated with BRED.

Phage (*gene*)	Mutation^1^	Substrate	# Mixed Plaques/Total analyzed (%)^2^		# Muts/Total (%)^3^
			Flanking PCR	Selective-PCR	
Giles (*20*)	Deletion	200 bp	3/29 (10.3)		0/8; 1/8 (6.2%)
Giles (*20*)	Deletion	200 bp	22/144 (15.3%)	53/144 (36.8%)^4^	11.2%^5^
Giles (*20*)	bp sub.	100 bp	1/18 (5.6%)^6^		3/10 (30%)^6^
Giles (*31*)	Deletion	200 bp	9/103 (8.7%)	30/105 (28.6%)^4^	1/100; 0/100 (0.5%)^7^
Giles (*29*)	Deletion	200 bp	3/59 (5.1%)		3/11 (27.3%)
Giles (*61*)	Deletion	200 bp	2/17 (11.8%)		2/8 (25%)
Giles (*32*)	His_6_ tag	218 bp		6/17 (35.3%)^8^	3/8; 1/8 (25%)^8^
Giles (*62*)	His_6_ tag	218 bp		4/8 (50%)^8^	1/8; 0/8 (6.2%)^8^
BPs (*44*)	Deletion	200 bp	2/17 (11.8%)		3/19 (15.8%)
BPs (*50*)	Deletion	200 bp	1/28 (3.6%)		3/19 (15.8%)
BPs (*52*)	Deletion	200 bp	3/5 (60%)		3/22 (13.6%)
BPs (*54*)	Deletion	200 bp	10/56 (17.9%)		5/19; 1/18 (16.2%)
BPs (*58*)	Deletion	200 bp	1/16 (6.3%)		2/22 (9.1%)
BPs (*54*)	Replace	942 bp^9^	2/22 (9.1%)	40/40 (100%)^10^	1/11; 0/16 (3.7%)
Halo (49)	Deletion	200 bp	4/37 (10.8%)		1/33; 0/13 (2.2%)
Halo (*52*)	Deletion	200 bp	1/8 (12.5%)		3/14 (21.4%)
TM4 (*29*)^11^	Nonsense	100 bp		2/18 (11.1%)^12^	2/100 (2%)^12^
Che9c (*61*)	Deletion	200 bp	2/16 (12.5%)		1/25 (4.0%)

1 Mutations generated were gene deletions, insertion of C-terminal His_6_ tags, nonsense mutations, or replacement of phage gene with *gfp* (Replace).

2 Plaques recovered from electroporation were analyzed by PCR as indicated.

3 Individual plaques were screened after re-plating of an initial mixed plaque. Values where plaques were recovered and tested from more than one mixed plaque are separated by a semicolon and combined for average percentage.

4 Plaques were screened by DADA-PCR.

5 A total of 195 individual plaques from ten initial mixed plaques were screened, three of which contained no mutants.

6 Plaques were screened by restriction digestion.

7 Plaques were screened genetically by complementation.

8 Plaques were screened with an upstream primer complementary to the tag sequence.

9 Substrate has 100 bp homology flanking BPs *gene 54*.

10 Primer is complementary to *gfp*.

11 BRED was performed in *M. smegmatis* mc^2^155∶pJV62 cells, which express Che9c gp61^20^.

12 Plaques were screened by MAMA-PCR.

### Generation of other mutations and application of BRED to other phages

BRED can also be used to construct insertions, replacements, and for the addition of gene tags (such as His_6_). We constructed two gene tags, one introducing a His_6_ tag onto the C-terminus of Giles gp32 (LysB), and a second introducing a His_6_ tag onto Giles gp62, a putative DNA methylase. In each case 218 bp dsDNA substrates were used, mutant-containing plaques were identified by PCR analysis of 18 individual plaques with a primer complementary to the His_6_ tag, and purified mutants were identified after re-plating and re-testing (Table 3). An insertion/replacement mutant was constructed similarly, but in the context of the mycobacteriophage BPs genome (unpublished). In this case, a substrate was generated by PCR amplification of a *gfp* gene cassette with 100 bp flanking sequence to target replacement of gene *54*. Following co-electroporation, mixed plaques were identified, and a homogenously pure mutant was readily recovered (Table 3). A notable observation in this construction was that by using highly selective PCR (with one of the primers annealing within *gfp*), we could confidently detect the mutant in every one of 40 plaques examined, reflecting a remarkably high level of mutagenesis.

The BRED strategy is broadly applicable to mycobacteriophages, and we have successfully manipulated the genomes of phages Giles, TM4, Halo, BPs and Che9c (Table 3). No substantial differences in frequencies were observed, with mutant-containing plaques occurring in no fewer than 5% of the primary plaques recovered when screening by flanking PCR and in no fewer than 20% when using a more sensitive PCR that preferentially amplifies the mutant (Table 3). In some examples, we have not yet been able to recover a purified mutant derivative, probably reflecting poor viability of the mutants. We therefore predict that all phages that can propagate in *M. smegmatis* will be suitable substrates for BRED mutagenesis. A summary of all mutant derivatives constructed is shown in Table 3.

## Discussion

We have described here a simple and facile method for mutagenesis and manipulation of mycobacteriophage genomes. The BRED strategy takes advantage of the ability to simultaneously introduce phage DNA and a targeting substrate into recombination-proficient *M. smegmatis* cells, such that a high proportion (>10%) of plaques recovered contain the desired mutant. The impressively efficient recombination enables the identification of mutants by two rounds of a small number of PCR reactions.

While both dsDNA and ssDNA substrates can potentially be used for recombineering, we generally favor 200 bp dsDNA when generating deletions or adding tags using BRED. These dsDNA substrates avoid potential complications of huge strand biases observed when recombineering the mycobacterial chromosome[28], and because we know little about mycobacteriophage DNA replication, the best strand to choose for recombineering cannot be easily predicted. In practice, generating a 200 bp substrate using a three-primer PCR strategy is simple, cheap, and effective for most BRED applications (Table 3).

BRED is a related strategy to a method described for recombineering bacteriophage λ[18]. In that approach, *E. coli* cells are infected with the λ phage, recombineering functions are induced, competent cells are prepared, the targeting substrate is introduced by electroporation, and plaques are recovered after completion of a lytic cycle. BRED differs from this in several critical respects. First, the λ system relies on a very high proportion of cells being competent to take up DNA by electroporation, and there are few bacterial systems that are as efficient as this. Second, the proportion of recovered mutants is relatively low (∼2%) and thus more difficult to detect using PCR. Third, because BRED involves recovery of plaques prior to lysis, non-viable mutants can be propagated with assistance of helper phage in a mixed plaque and then recovered by complementation or suppression. BRED is expected to be applicable to phages of other bacterial hosts in which recombineering systems have been described, including pathogenic *E. coli* [34], *Shigella* [35], *Salmonella enterica* [36], and *Yersinia pseudotuberculosis* [37].

There appear to be few limitations to the application of BRED to mycobacteriophage genetics. We have manipulated several different types of phages and have introduced many different types of mutations. The largest deletion constructed thus far is the 1,173 bp deletion of Giles *lysA*, but larger deletions should also be possible provided that no essential functions are removed and that the mutant genome can be packaged. Likewise, the largest insertion/replacement we have created is the 750 bp *gfp* insertion into BPs (replacing gene *54*), and larger insertions should be possible provided that downstream genes expression is not impeded. Classes of mutants we do not expect to isolate are those losing *cis*-acting sites (such as the origin of replication) and dominant negative mutants.

Numerous potential applications of the BRED technology can be envisaged. First, since phage genomes are replete with genes of unknown function, these can be systemically deleted to test if they are required for phage growth; moreover, precise deletions can be constructed to avoid genetic polarity. Second, protein extensions such as His_6_ or StrepII affinity tags can be readily introduced for interactome investigations of phage-infected cells. Third, reporter genes can be inserted at precise genomic locations, either to examine gene expression patterns or for use in diagnostic applications[23], [24]. Additionally, unique restriction sites can be introduced to create mycobacteriophage cloning vectors and for constructing phage chimeras.

BRED has the potential to substantially alter the field of bacteriophage genetics. It offers the prospects of moving beyond genomic descriptions of novel genes and genomes and making phage biology accessible to functional genomics. It should also enable a systems-wide characterization of bacteriophages and an understanding of their molecular circuitry in an integrated manner.

## Methods

### 

#### Bacterial strains and media

*M. smegmatis* mc^2^155[38], the recombineering strain containing plasmid pJV53[27] that expresses Che9c genes *60* and *61* under the control of the inducible acetamidase promoter[32] and the suppressor strains[33] have been described previously. Plasmid pKMC4 (K. Payne, unpublished data) contains the Corndog *lysA* gene under control of the acetamidase promoter. Strains were grown on Middlebrook 7H10 medium supplemented with 10% (Albumin Dextrose Complex) ADC and 0.05% Tween 80, as described previously[27], although Tween was omitted and 1 mM CaCl_2_ included for phage infections.

#### Construction of recombineering substrates

Recombineering substrates were constructed as described previously[25]. For deletions, a 100-base oligonucleotide (50 bp of upstream and downstream homology) and two flanking 75-base primers (each complementary to 25 bases at each end of the 100-mer) were designed, and the final 200 bp product was amplified by PCR; substrates introducing His_6_ tags were constructed similarly. To insert the *gfp* gene, two 75-base primers were used to amplify *gfp* from plasmid pMN437 (a generous gift from Michael Niederweis), with 25 bases complementary to each end of *gfp* and 50 bp of homology upstream and downstream of the inserted sequence. The PCR product was further extended by a second round of PCR to add an additional 50 bp of homology to each end (to generate a substrate with 100 bp homology on each end). All oligonucleotides were purchased from IDT Inc. and were gel purified; these are listed in Table S1. PCR products were processed using QIAquick PCR-Purification (QIAGEN) or MinElute PCR Purification Kits (QIAGEN), eluting DNA in a minimal volume of sterile water.

#### Bacteriophage Recombineering of Electroporated DNA (BRED) in *M. smegmatis*

Induced electrocompetent *M. smegmatis* mc^2^155:pJV53 cells were prepared as described previously[27]. Briefly, after growth to OD_600_ of ∼0.4 in Middlebrook 7H9 with 0.2% glycerol, 0.05% Tween 80, and 0.2% succinate, cells were induced with 0.2% acetamide, grown for 3 hours, washed three times with ice-cold 10% glycerol, and stored at −80°C. Aliquots (100 µl) were co-electroporated with phage DNA and recombineering substrate, recovered at 37°C in 7H9 containing 10% ADC and 1 mM CaCl_2_ for ∼2 hours (lysis does not occur until after 3 hours), and plated on 7H10 agar as top agar lawns with approximately 300 µl of *M. smegmatis* mc^2^155.

Plaques were picked into 100 µl phage buffer (10 mM Tris-HCl, pH 7.5; 10 mM MgSO4; 68.5 mM NaCl; 1 mM CaCl_2_). One microliter was PCR amplified with flanking primers (25–35 bp) annealing upstream and downstream of the mutant allele, or by Deletion Amplification Detection Assay (DADA)-PCR using Platinum Taq High Fidelity DNA Polymerase (Invitrogen) and an upstream primer whose 3′ end anneals over the deletion junction. DADA-PCR parameters were similar to those described for MAMA-PCR[30], with the combined annealing and extension step performed at or just above the melting temperature of the DADA-PCR primer. Plaques containing mixtures of deletion and wild-type DNA were picked into 100 µl buffer, and 10 µl of 10^−3^, 10^−4^ and 10^−5^ dilutions were plated with 300 µl *M. smegmatis* cells. Either individual plaques from the 10^−4^ and 10^−5^ plates or lysates from 10^−3^ or 10^−4^ plates were screened for the presence of the mutation by PCR as described above.

#### Complementation of the Giles lysAΔ

Cultures of mc^2^155 containing pKMC4 (complementation) or pLAM12 (control) were grown to OD_600_ 1.0 in 7H9 supplemented with 0.2% glycerol, 0.05% Tween 80, and 0.2% succinate; cells were pelleted and resuspended in one-half volume of the same medium without Tween 80. Approximately 500 µl aliquots were infected with 10 µl of serial phage dilutions, adsorbed at room temperature for 30 minutes, and plated as top agar lawns with 0.2% acetamide. Plaques from the complementation strain were replica-picked onto top agar lawns with either the complementation strain or the control strain to identify a complementation-dependent mutant plaque.

## Supporting Information

Table S1 (0.13 MB DOC)
