# Supplementary material for: BRED: A Simple and Powerful Tool for Constructing Mutant and Recombinant Bacteriophage Genomes
Source: PLoS One. 2008 Dec 17;3(12):e3957. doi: 10.1371/journal.pone.0003957 (PMC2597740; doi:10.1371/journal.pone.0003957)
Supplement: Table S1 — (0.13 MB DOC) [file pone.0003957.s001.doc]

Table S1. Oligonucleotides used for BRED mutagenesis

| **Name** | **Sequence** | **Use** |
| --- | --- | --- |
| LJM95 | GGCCGATGTGATGATCCCGAACTACAACACCCCGCA | Giles *gene* *20* muts^FP^ |
| LJM96 | ATTCGGCCTGCGCGATGGCATCCCAATCGGGCA | Giles *gene* *20* muts^FP^ |
| LJM97 | CGTCGGCCTTCGCCGCGTCCAACTGCCGT | Giles *gene* *20* muts^FP^ |
| LJM116 | GACGGTGCCCGATTGGGATGCCATCGCGCAGGCAGCTTCCGGCGGCAACTGGGCGATCAACACCGGCAAC | Giles *gene* 20 Base sub. |
| LJM117 | GTTGCCGGTGTTGATCGCCCAGTTGCCGCCGGAAGCTGCCTGCGCGATGGCATCCCAATCGGGCACCGTC | Giles *gene* 20 Base sub. |
| LJM119 | TTGCCACCGGCCGGGCGTTGGGGATCCTCCAATCGTTCGCTAACGGCGGGTTGAACACCGGCGCGGCGACGCAGACGGTGCCCGATTGGGATGCCATCGC | Giles *gene* 20 402 bpΔ |
| LJM120 | GCGATGGCATCCCAATCGGGCACCGTCTGCGTCGCCGCGCCGGTGTTCAACCCGCCGTTAGCGAACGATTGGAGGATCCCCAACGCCCGGCCGGTGGCAA | Giles *gene* 20 402 bpΔ |
| LJM121 | CGAAGCGTTTATCCCGATCAACGGGTCGCAACGGTCGAAGGACATCTGGGTTGCCACCGGCCGGGCGTTGGGGAT | Extend LJM119 |
| LJM122 | AGTACCCGTTGCCGGTGTTGATCGCCCAGTTGCCGCCGGATTCGGCCTGCGCGATGGCATCCCAATCGGGCACCG | Extend LJM119 |
| LJM123 | GACATGCAGGCACTGTTGCAGCAGTACCCGATG | Giles *gene* 20 muts^FP^ |
| LJM124 | TTGCCACCGGCCGGGCGTTGGGGATCCTCCAATCGTTCGCTAACGGCGGGATCGACCCGGAGACCGGCGAGCGCGGGTTCTACACCCCGGACCCGAAGAA | Giles *gene* 20 717 bpΔ |
| LJM125 | TTCTTCGGGTCCGGGGTGTAGAACCCGCGCTCGCCGGTCTCCGGGTCGATCCCGCCGTTAGCGAACGATTGGAGGATCCCCAACGCCCGGCCGGTGGCAA | Giles *gene* 20 717 bpΔ |
| LJM129 | AGTACCCGTTGCCGGTGTTGATCGCCCAGTTGCCGCCGGAAGCTGCCTGCGCGATGGCATCCCAATCGGGCACCG | Extend LJM119 incl. bp sub. |
| LJM130 | GTGGATCCGCCGGTATGCCGGTGTACGCCATCCAATCCGGCACCGTCATCCCGCTCACGGCCGCGAAGATTGGGTTGGTGCTCTGATGGCGTGGAAACCT | Giles *gene* 31 *lysA*Δ |
| LJM131 | CACGACCGGCCGGTCCGCCCCGCACGGCGGCGTTGACTTCGGCCGGGCCGGTGGATCCGCCGGTATGCCGGTGTA | Extend LJM130 |
| LJM132 | TCCTTCGCGTCGGCGATGTCGGGGTGCCGATCTCCGATTTGGTATTCGGTAGGTTTCCACGCCATCAGAGCACCA | Extend LJM130 |
| LJM133 | CCAGCTTCTGTACCGCGACTAGCGCGTTC | Giles *lysA*Δ ^FP^ |
| LJM134 | CACGCCTGCAACACCCGCGACACGTAGTC | Giles *lysA*Δ ^FP^ |
| LJM135 | TACGCCATCCAATCCGGCACCGTCATCCCG | Giles *lysA*Δ ^DP^ |
| LJM136 | GATCCTCCAATCGTTCGCTAACGGCGGGTTGAA | Giles *gene* 20 402 bpΔ^DP^ |
| LJM137 | GTACCGGTAGACCGTTCGGCAGCGTTTGG | Giles *gene* 20 402 bpΔ^FP^ |
| LJM144 | AGAGACTGGGCCGGGCGCACCCCGGCCGTCACCGCACACCACCACCACCACCACTAACACACCGAAGGGATCGCAACAGTGAGCACGAAT | Giles *gene* *32*-His_6_ |
| LJM145 | TACGAATTCCGCGAAGTGTGGCCCGGTCAAACGTATCTGGGCCTGGCCATCCAACACGTCAGAGACTGGGCCGGGCGCACCCCGGCCGTC | Extend LJM144 |
| LJM146 | GAGCGTGCGTAGCGGATTGACACCCGGCGCGGGGACCGTGGCCGGGTTCGGATTGGGCGGATTCGTGCTCACTGTTGCGATCCCTTCGGT | Extend LJM144 |
| LJM147 | CCGTCACCGCACACCACCACCACCAC | Giles *gene* *32*-His_6_^TAG^ |
| LJM148 | CATCCCCTGCGGTGCCGGTTCGTCG | Giles *gene* *32*-His_6_^FP^ |
| LJM149 | GCCAACCGGTTGTCGCAGATGTGCCTGGGGTTCGCACACCACCACCACCACCACTGAAGTCCGGCGACGGCACGGTGAGGGGACACCGCC | Giles *gene* *62*-His_6_ |
| LJM150 | CGCAACCTGGGGCGCAAAGCAATCGGCGTGGAGATAGACGAACGGTATTGCGAAGTGATCGCCAACCGGTTGTCGCAGATGTGCCTGGGG | Extend LJM149 |
| LJM151 | TGCACGACGGGCACGGCCGTGTGAGGGCGTCGCGCACCGCCGGGTCGGCGCGGTTCTCCAGGCGGTGTCCCCTCACCGTGCCGTCGCCGG | Extend LJM149 |
| LJM152 | GCCTGGGGTTCGCACACCACCACCAC | Giles *gene* *62*-His_6_^TAG^ |
| LJM153 | GGGCGTGTTCCGTGTCGTGTCCGTGAC | Giles *gene* *62*-His_6_^FP^ |
| gp29- IntΔ | ACCCCCGAGAGGACACCGCAATGGGGCGACGCGGCAACCGCGAGGCACACGGATCGCGGACGGCAGCGGACAGTCTTAGCGAGCTCGACGGACGCGGAC | Giles *gene* *29* *int*Δ |
| gp29-Ext1 | CACCTATCCCGACCGGGCACTATTCGGCGTGGATCCCCACGCACCCGGCGACCCCCGAGAGGACACCGCAATGGG | Extend gp29-IntΔ |
| gp29-Ext2 | GCCCCTTTTTCTGTTTGACCCCAATTTTGACCCCAATTGCTCCGGCGTCCGTCCGCGTCCGTCGAGCTCGCTAAG | Extend gp29-IntΔ |
| gp29F | GAACGCGCTAGTCGCGGTACAGAAGCT | Giles *gene* 29Δ^FP^ |
| gp29R | ACCCCAATTGCATCGGGGTTCGGGGC | Giles *gene* 29Δ^FP^ |
| gp61Δ | CACACCCGCGACAACATCCGGGGCCGGTGATGGACGCCCCGCTGTTGTTCATCACCGAACGGGCGGCCCGGTGATGGACGAAGTGTGCGTGCACTGCGGT | Giles *gene* 61Δ |
| gp61-Ext1 | ATCCGACGGTTGAGGTCGATGAACCGGCCGACATCGGCGCACCGACGCTGCACACCCGCGACAACATCCGGGGCC | Extend gp61Δ |
| gp61-Ext2 | TGCACCAACGCCGCACCGCCCCACGGCGTCCGAACGACGTGGACCGGCCGACCGCAGTGCACGCACACTTCGTCC | Extend gp61Δ |
| gp61F | CGAATCACGTTGACCGCCATGCCCGGA | Giles *gene* 61Δ^FP^ |
| gp61R | TCCCGTAGGGCGGGTCGGTTATCAG | Giles *gene* 61Δ^FP^ |
| gp44Δ | GCTGCGGATCCCACCAGTGAAGGGAACGAGTGACCATGGGACTGATTCAGGGCGGCAAGTGAGCACGGCAGACGCCGGCCACTCGGCGCACTGGATCAGC | BPs *gene 44Δ* |
| gp44Δ-Ext1 | CCGGCATTCTCTCGGACGCTGCCCGCGCCGAGTCCGAATCCGTGGCGCCCGCTGCGGATCCCACCAGTGAAGGGA | Extend BPs gp44Δ |
| gp44Δ-Ext2 | CCGGCCGTCTCGGGTGAGTCGCGGAACATCGCGAAGCACGCCGCGCACTCGCTGATCCAGTGCGCCGAGTGGCCG | Extend BPs gp44Δ |
| gp44F | GACGTCCTCATGGGTATCAGCTACA | BPs *gene* 44Δ^FP^ |
| gp44R | GACGTCCTCATGGGTATCAGCTACA | BPs *gene* 44Δ^FP^ |
| gp50Δ | GAGCTCGTCGCCGAGGACCAGGAGGCCGGCCGATGACCGCCCGAAACGAGAAGGGACACCGCGCATGATCGCCGTCGGAATCGACCCGTCACTGACCTCG | BPs *gene 50Δ* |
| gp50Δ-Ext1 | TGGCGCGATCGCGCGAAGCGCGCCGGCACCCGCACCGCGCGGCCGGCGACGAGCTCGTCGCCGAGGACCAGGAGG | Extend BPs gp50Δ |
| gp50Δ-Ext2 | TATCGGCCGTAGTGGGCGAGCTTCCCGTCGACGAGCACGGCGACGCCGGTCGAGGTCAGTGACGGGTCGATTCCG | Extend BPs gp50Δ |
| gp50F | TACGTAGGTAAGGACAACCCGATCC | BPs *gene* 50Δ^FP^ |
| gp50R | ACTTCATCGGAACTGCGCTTTCTTC | BPs *gene* 50Δ^FP^ |
| gp52Δ | GTGGCCGAAGAAAGCGCAGTTCCGATGAAGTGCCCGAGCTGCGGCGCCTAGGGGTGAACGCATGAGCTGGGAACCGATTCTGGCCGCGCTCGGCGGATAT | BPs *gene 52Δ* |
| gp52Δ-Ext1 | CTGCCGTTCGAGCCGAAGGAACGCCACCGCACGGCGTTAGCCTTGCTGCCGTGGCCGAAGAAAGCGCAGTTCCGA | Extend BPs gp52Δ |
| gp52Δ-Ext2 | CATTCGGACTGCATCACGGCCCGGAACACGTCGCGGCCGGCGAAGAACAGATATCCGCCGAGCGCGGCCAGAATC | Extend BPs gp52Δ |
| gp52F | AGGACGACGAGACATGTCCACAC | BPs *gene* 52Δ^FP^ |
| gp52R | AAGATCAGCTCGTGACCGTCTT | BPs *gene* 52Δ^FP^ |
| gp54Δ | AAGTGATGTCCCGAATCTGGGGAGCGCTGCCGCTACTCGACCCGATGGACGCGCCGATGCCCAGATACACGGGGTAGCGGCTGTTCGTCGCTCTGCGGGC | BPs *gene 54Δ* |
| gp54Δ-Ext1 | TCCTGGCCGCGGACCTACGGGCGATCGGCCGAGCACAACGGCGAAAGGCGAAGTGATGTCCCGAATCTGGGGAGC | Extend BPs gp54Δ |
| gp54Δ-Ext2 | TCGCAAGCGTTCACCATGTCCGATACCGGAATATTCCCGCGCCCTCAGCGGCCCGCAGAGCGACGAACAGCCGCT | Extend BPs gp54Δ |
| gp54F | GAACCCGCAACGGATCATGTTCGGGCTCGC | BPs *gene* 54Δ^FP^ |
| gp54R | GCTCGTTCTGGTGGGCTTCGATGCGGAT | BPs *gene* 54Δ^FP^ |
| gp58Δ | CCGATTACCGACACTCGTAGGGAGATGACGACTATGCCGACCACAGAGCATGGTTGTTTTCGATTACGACACCTCGCCCAACAAGGCACAAGCGGATACA | BPs *gene 58Δ* |
| gp58Δ-Ext1 | CGTCGCCGTTTCTCCAGGTAGGCAGCCGATCTGACAGCGCACACATGTTTCCGATTACCGACACTCGTAGGGAGA | Extend BPs gp58Δ |
| gp58Δ-Ext2 | TGCGGGCGCCGTTCTGGTCGGTCTCGACGTTATCTGGGGTCATCGTCAGCTGTATCCGCTTGTGCCTTGTTGGGC | Extend BPs gp58Δ |
| gp58F | GAGCTCTACACGTCCGCTCACT | BPs *gene* 58Δ^FP^ |
| gp58R | AAACCTCCTGGTCGTAGAAACAAAC | BPs *gene* 58Δ^FP^ |
| LMO01 | CCGCGGACCTACGGGCGATCGGCCGAGCACAACGGCGAAAGGCGAAGTGATTAATTAACAGAAAGGAGGTTAATA | *gfp* + 50 bp upstream BPs *gene 54* |
| LMO02 | TACCGGAATATTCCCGCGCCCTCAGCGGCCCGCAGAGCGACGAACAGCCGATCTACTTGTACAGCTCGTCCATGC | *gfp* + 50 bp downstream BPs *gene 54* |
| LMO03 | CGTGTTCCGGGCCGTGATGCAGTCCGAATGGATCTTCGGCCGGATCCTGGCCGCGGACCTACGGGCGATCGGCCG | Adds 50 bp upstream BPs *gene 54* |
| LMO04 | ACAAGTGTCACACATTGGCGACATATGTCGCAAGCGTTCACCATGTCCGATACCGGAATATTCCCGCGCCCTCAG | Adds 50 bp downstream BPs *gene 54* |
| LMO05 | GCATGAGCTGGGAACCGATTCTGGC | BPs *gene* 54-gfp^FP^ |
| LMO06 | CATTGGCGACATATGTCGCAAGCGTTCAC | BPs *gene* 54-gfp^FP^ |
| LMO08 | CGTCAACGGCCACAAGTTCTCCGTC | *gfp* primer |
| gp49Δ | TATCACTGCCCCACCCACCAGAAAGGCACCCGATGACCCTCCAAACGCCACAGGAGGCCGGCCGATGACCGCCCGAAACGAGAACGTCGCGATCTTCAAC | Halo *gene 49Δ* |
| gp49Δ-Ext1 | TGCCGAGCGAACGCGGGGCGCCCTGGCAGTACCGACCAGCCACCCGCACGTATCACTGCCCCACCCACCAGAAAG | Extend Halo gp49Δ |
| gp49Δ-Ext2 | GTGCCGTCGGGCCCGTCGGGGTGCCATGCCGGAAGCGGCAGGACAAGCGGGTTGAAGATCGCGACGTTCTCGTTT | Extend Halo gp49Δ |
| gp49F | CGACTTTCGAGACGCACAATCTT | Halo *gene* 49Δ^FP^ |
| gp49R | GAGGTCAGTGACGGGTCGATTC | Halo *gene* 49Δ^FP^ |
| gp52Δ | GCGTTAGCCTTGCTGCCGTGGCCGAAGAAAGCCCAGGTCCGATGATGACCCCCGCATGAGCACACCAGGACGGCGACCAGTGAGCCGCCGGCGCTCGTTC | Halo *gene 52Δ* |
| gp52Δ-Ext1 | TCAAGCTCGGCGACCCGCTGCCGTTCGAGCCGAAGGAACGCCACCGCACGGCGTTAGCCTTGCTGCCGTGGCCGA | Extend Halo gp52Δ |
| gp52Δ-Ext2 | GCGGCGTGGATCTTGGCGCCGAGCTCTTCGATTTCGGCGAAGTGCTCGTCGAACGAGCGCCGGCGGCTCACTGGT | Extend Halo gp52Δ |
| gp52F | GCATGGCATCTACGGCAATCTC | Halo *gene* 52Δ^FP^ |
| gp52R | GTGCTCAGGTTCGTGCTGTTCG | Halo *gene* 52Δ^FP^ |
| gp29Q/TAG1 | CGACAAGCGCGGCGCCTGCGTCATTGCGGGCATGACGATTTCCTAGGAGGTCGGCGTAAAGGACAACGACCCGCCGTTCGAGCGGCGGTT | TM4 *gene* 29 nonsense mutant |
| gp29Q/TAG2 | AACCGCCGCTCGAACGGCGGGTCGTTGTCCTTTACGCCGACCTCCTAGGAAATCGTCATGCCCGCAATGACGCAGGCGCCGCGCTTGTCG | TM4 *gene* 29 nonsense mutant |
| gp29-F | GGCATGACGATTTCCTAG | TM4 *gene* 29^MP^ |
| gp29-R | ACATAGGCGTCGTATTGC | TM4 *gene* 29^FP^ |
| JCV399 | CGAAGAGGCCGACCCGATTGAAGGGGATTACATCTATGGCTGAAAATGCTGGGCCCAACGCATGAGCGCCCCGGCGAACCACGACGCGGTGGTTGATCTG | Che9c *gene* 61Δ |
| JCV400 | ACCGTCGGCGACGTCGTCACCGACAGCTATATCTACGACACCGACCCGCTCGAAGAGGCCGACCCGATTGAAGGG | Extend JCV399 |
| JCV401 | GGCCTCGGCGTAGTGGTACGTCGGCGGGCGCGTCATGACTGCACCACCTGCAGATCAACCACCGCGTCGTGGTTC | Extend JCV399 |
| JCV402 | GACGTGGTCACGATCAGCCTGCCCT | Che9c *gene* 61Δ^FP^ |
| JCV403 | CAACTTGAACGCGATCGCGGGCACG | Che9c *gene* 61Δ^FP^ |

^FP^Flanking primer.

^DP^DADA-PCR primer.

^TAG^Primer specific to the tag sequence.

^MP^MAMA-PCR primer.
